# What does valve, not myocardial LGE mean? Could it portend post-MI emergence of mitral regurgitation?

**DOI:** 10.1186/1532-429X-16-S1-P98

**Published:** 2014-01-16

**Authors:** Hari Bogabathina, Mark Doyle, Ronald B Williams, June A Yamrozik, Diane V Thompson, Robert W Biederman

**Affiliations:** 1 Cardiac MRI, Allegheny General Hospital, Pittsburgh, Pennsylvania, USA

## Background

Post-MI mitral regurgitation is thought to be due to passive, rather than active remodeling of mitral valve apparatus and relationship with other cardiac structures contributing to mitral regurgitation. Standard CMR late gadolinium enhancement (LGE) may be sensitive to non-myocardial pathology involving the mitral valve leaflets.

## Methods

Presence or absence of MVE was noted in patients presenting for routine CMR with MI and non-MI indications requiring LGE. Chi square analysis was performed for non-contiguous variables and SPSS, Chicago software was utilized for statistical analysis. We hypothesize the presence of mitral valve enhancement (MVE) on LGE imaging in post-MI patients is associated with an increased incidence of MR.

## Results

**Figure 1:**
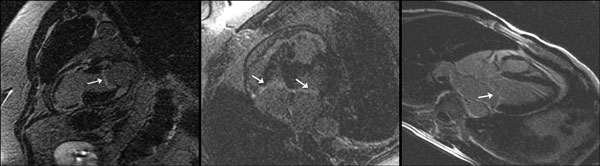
**Examples of MVE**.

Patients (87; M = 54, F = 33) underwent LGE-CMR studies utilizing 1.5T GE (Milwaukee, WI) scanner with MultiHance (Bracco, Princeton, NJ) gadolinium contrast administration. LGE+ (present) in 68 and LGE- (absent) in 19 studies. Post-MI patterns of LGE+ in 51 and LGE- in 36 pts. MVE+ in 39, MVE- in 48. MR+ present in 67 and MR- in 20 studies. MVE was observed chiefly in post-MI patients (33 of 51; 65%) and infrequently in non-post-MI patients (6 of 36; 17%); χ2 = 17.8, p < 0.001, power = 0.995. Further, MR was present more frequently in patients with MVE (36 of 39; 92%) compared to patients without MVE (31 or 48; 65%); χ2 = 7.8, p = 0.005, power = 0.814 (see Figure 1).

## Conclusions

MVE is present in a large number of post-MI patients but rarely in non-post-MI patients. Post-MI patients with rather than without MVE are far more likely to have MR. These observations suggest a specific, as yet unknown, reactive process may contribute to mitral leaflet remodeling in post-MI patients potentially contributing to the increased incidence of MR in post-MI patients. Thus, active remodeling of the mitral valvar structure may be operative in conjunction with passive geometric LV remodling and *collectively *promote post-MI mitral regurgitation

## Funding

Internal.

